# Beneath the smoke: Understanding the public health impacts of the Los Angeles urban wildfires

**DOI:** 10.1097/EE9.0000000000000388

**Published:** 2025-04-28

**Authors:** Tarik Benmarhnia, Nicole A. Errett, Joan A. Casey

**Affiliations:** a Scripps Institution of Oceanography, University of California, La Jolla, California; b University of Rennes, Inserm, EHESP, Irset (Institut de recherche en santé, environnement et travail), Rennes, France; c Environmental and Occupational Health Sciences, University of Washington School of Public Health, Washington; d Epidemiology, University of Washington School of Public Health, Seattle, Washington

The devastating wildfires across Los Angeles (LA) caused unprecedented damage to urban communities and infrastructure. These fires are a preview of the coming decades as climate change intensifies. The winter of 2023/2024 brought extreme precipitation, resulting in rapid vegetation growth. This was followed by hot temperatures in the summer and fall of 2024, which, combined with a lack of precipitation, dried out this vegetation. When paired with intense Santa Ana winds in January, this created the perfect circumstances for large wildfires.

The largest Californian wildfires typically ignite in remote and unpopulated areas, though fires have affected California’s urban infrastructure.^1,2^ For example, it has been estimated that the 2018 Camp Fire destroyed 18,804 structures after burning over 153,000 acres in Butte County. There were at least seven wildfires in LA in January, including the Eaton and Palisades fires. The Eaton fire burned 14,000 acres and damaged more than 7000 structures. Property damage from the Palisades and Eaton fires has been estimated to top $35 billion, the most material damage by an extreme weather event in US history.^3^

Wildfire smoke has been identified as a significant driver of public health burden, and the LA Fires also present unprecedented features that may require a change in how air quality is monitored and regulated in fire-prone areas. In addition, these fires constitute an opportunity to highlight critical and less-documented public health challenges and evidence gaps for enhancing preparedness and long-term resilience among affected communities and describe health impacts beyond environmental contamination. We also highlight the environmental justice implications because, as with nearly every disaster, some subgroups will fare much worse than others.

## The role of smoke

Large urban wildfires cause similar impacts on population health as other wildfires but also have unique impacts. Communities directly impacted by a fire can be exposed to various chemicals through the combustion of buildings, plastics, electronics and appliances, vehicles, and artificial structures.^4^ Such chemicals vary depending on the combustion source but can include heavy metals (e.g., lead), polybrominated diphenyl ethers (PBDEs), per- and polyfluoroalkyl substances (PFAS), and other known carcinogens such as polycyclic aromatic hydrocarbons (PAHs) and asbestos.^5,6^ In the case of the LA Fires, nearby residents were also exposed to a flame retardant called Phos-Chek,^7^ which may be enriched with heavy metals.^8^ Further, smoke from wildfires that burned structures appears enriched in heavy metals compared with wildfires that burned vegetation alone.^9^ While some of these substances cannot travel long distances, others can as part of smoke. Smoke drives most of the health burden of wildfires due to broad population exposure. The directly attributable death toll of the LA Fires is 25–30 individuals, typically due to acute intoxication by carbon monoxide. These estimates represent just a small proportion of the full public health burden.

Wildfire smoke includes several components, including fine particles (PM_2.5_), toxic chemicals, and microorganisms. Smoke can travel hundreds of miles or farther, as demonstrated by the 2023 wildfires in Canada,^10^ thus exposing millions. Recent health impact assessments estimate the burden attributable to wildfire PM_2.5_ to be substantial. It has been estimated that 85 direct fatalities resulted from the 2018 Camp Fire. Though most studies have focused on short-term impacts, the public health burden likely has a long tail.^11,12^ One study estimated that the 2018 wildfire smoke events in California may have led to as many as 12,000 premature deaths when considering multiple years after exposure.^13^

Thresholds in air quality indices derived from federal regulatory standards drive the issuance of air quality alerts and adoption of local preventive actions but do not currently consider the specific source of PM_2.5._ Some animal^14,15^ and human^16^ studies suggest that wildfire smoke PM_2.5_ may have a higher toxicity than PM_2.5_ from other sources, such as traffic. It may, therefore, be necessary to revisit air pollution monitoring during wildfires by adjusting alert thresholds if the presence of smoke is suspected. More research is also needed about the detailed composition of urban wildfire PM_2.5_ and understanding the dynamics of the transport of this PM_2.5_.

## Evacuation

Evacuation itself leads to substantial health impacts at short and longer time scales. Those evacuating face extreme stress and impacts on mental health, even years after the events.^17^ The exact number of evacuees from the LA Fires remains unknown, but more than 150,000 people were under evacuation notice 1 week after the fires started. Even when homes are not damaged or destroyed, evacuation disrupts multiple dimensions of people’s lives, including work, education, community gatherings, and health care access. Delayed and disrupted healthcare, including prescription drug and opioid treatment program access,^18^ has been documented after hurricanes, floods, and wildfires. It is thus particularly important to identify which individuals and communities are particularly at risk of developing severe mental or physical health conditions in the months following wildfires. By leveraging data from previous disasters and electronic health records, we could design vulnerability profiles to identify who to target for preemptive screening for disease onset or exacerbation of existing comorbidities. This could help optimize the allocation of resources during the post-fire period.

## Power outage

While the causes of the LA Fires are still under investigation, the power grid is a prime suspect, especially for the Eaton and Hurst Fires. The risk of the grid sparking a fire increases with downslope Santa Ana winds, and wind likely strained the power grid preceding the LA Fires’ ignition.^19^ Between 2016 and 2020, power lines caused six of California’s 20 most destructive wildfires,^20^ and we recently found an increasing trend in power outages co-occurring with wildfires in California.^21^ Utility companies have begun to employ Public Safety Power Shutoffs (PSPS), temporarily turning off power to specific areas to reduce the risk of electric infrastructure igniting wildfires or worsening those already burning. Millions were without power in Southern California in January 2025, many due to PSPS events.^22^ Such shutoffs may have unintended consequences, including reducing access to healthcare and de-energizing electricity-dependent durable medical equipment. Santa Ana winds can also cause off-season heat waves.^23^ Without power, people cannot run air conditioning to handle heat or high efficiency particulate air filters to reduce smoke exposure. Government officials and utilities should consider continuing to underground power lines in high wildfire-risk regions^24^ and weigh the benefits and costs of PSPS events for population health.

## Environmental injustice

Communities across LA will not experience the short- and long-term impacts of the fires equally. More advantaged individuals can flee or filter their way to lower exposure.^25^ Wildfires can amplify existing disparities in indoor air quality.^26^ Disparities in response will occur due to underlying health status, healthcare access, cumulative environmental burden, systemic racism, and social and financial resources.^27^

Inequalities persist and often increase after a disaster. Cascading events may lead to fractured communities, disrupted employment, education, and healthcare, potentiating chronic mental health issues. Certain groups, such as unhoused individuals and people who use drugs (PWUD), who already face greater health risks than others, are at particular risk.^28^ After Hurricane Sandy, 10% of PWUD reported no methadone access in the following, and 23% accessed treatment via a different program. While LA prepares for the postdisaster phase, social safety net measures to support PWUD and prevent an increase in individuals being unhoused are critical.

For people directly impacted, losing their homes and everything inside can be devastating. Federal support will not come close to covering the economic costs, and it is still unclear how the insurance claim process will unfold for those who did not have insurance canceled before the fires. For owners and renters, getting back to permanent housing may take a significant amount of time, and entire communities will be displaced for long periods, if not permanently. Fractured communities can lead to long-term isolation and other mental health impacts. As learned during prior disasters,^29^ community-based interventions that bolster social networks and cohesion can protect against deleterious mental health consequences. Those in burn or evacuation zones will experience more than housing loss, including probable increases in financial and food insecurities and short- or long-term loss of employment and health insurance. For those without wealth, such losses can quickly and adversely impact health.

## The return home: post-disaster consequences

The impacts of the LA Fires will extend beyond their containment dates, but we lack good evidence on possible postdisaster consequences for public health. For those able to return to smoke-damaged homes, indoor concentrations of volatile organic compounds may remain elevated for months.^30^ Those who can return to home may find their neighborhoods unrecognizable and without access to critical support (including social networks, healthcare, schools, and essentials like groceries).^31^ Infrastructure (e.g., water or power) may be disrupted for months, or even years. Others will not be able to return home, and finding a new place to live will not be easy. Rent prices have already skyrocketed in LA as of January 2025. Disaster-induced housing demand, particularly in environments with low supply, can lead to postdisaster gentrification, especially in the absence of housing support for renters and lower-income households.

Large surfaces burned, meaning toxic chemicals can penetrate damaged water pipes and water sources and contaminate the drinking water for large populations over an unknown time period.^32^ Unlike for other types of disasters, neither filtering nor boiling the water will help remove these harmful substances. Burnt areas are particularly prone to flash floods and landslides, as observed during the 2018 Camp Fire. While precipitation may appear as a resolution, a near-term atmospheric river event could devastate LA.^33^

Social disadvantage permeates the experience of extreme events, like the LA Fires, from preparation to response to recovery,^34^ concentrating burden among poorer communities and communities of color. Fast, targeted, and equity-oriented preparation and response can buffer against the worst health impacts. A robust and coordinated scientific response can support our understanding of long-term impacts and the effectiveness of approaches to minimize them. As fire risk in Southern California will continue to increase with climate change,^35^ these fires provide a window into the future and an opportunity to learn and ready ourselves for the next event.

## Conflicts of interest statement

The authors declare that they have no conflicts of interest with regard to the content of this report
